# Sex-Specific Plasma Metabolomic Signatures in COPD Reveal Creatine, Purine/Urate, and Bile-Acid Axes

**DOI:** 10.3390/metabo16030178

**Published:** 2026-03-07

**Authors:** Carme Casadevall, César Jessé Enríquez-Rodríguez, Alexandra Eliassaf, Ady Castro-Acosta, Rosa Faner, José Luis López-Campos, Eduard Monsó, Sergi Pascual-Guàrdia, Ramon Camps-Ubach, Borja G. Cosío, Alvar Agustí, Ori Shalev, Joaquim Gea, on behalf of the BIOMEPOC group

**Affiliations:** 1 MELIS Department, Universitat Pompeu Fabra, 08003 Barcelona, Spain; carme.casadevall@upf.edu (C.C.); cesarjesse.enriquez01@alumni.upf.edu (C.J.E.-R.); spascual@hmar.cat (S.P.-G.); ramon.camps.ubach@hmar.cat (R.C.-U.); 2 Hospital del Mar Research Institute, Servei de Pneumologia, Hospital del Mar, 08003 Barcelona, Spain; 3 Centro de Investigación Biomédica en Red, Área de Enfermedades Respiratorias (CIBERES), Instituto de Salud Carlos III(ISCiii), 28029 Madrid, Spain; rfaner@recerca.clinic.cat (R.F.); josel.lopezcampos.sspa@juntadeandalucia.es (J.L.L.-C.); eduardmonsomolas@gmail.com (E.M.); borja.cosio@ssib.es (B.G.C.); aagusti@clinic.cat (A.A.); 4 Metabolomics Unit, Core Research Facility, Faculty of Medicine and Hadassah University Hospital, The Hebrew University of Jerusalem, Jerusalem 91010, Israelori.shalev@mail.huji.ac.il (O.S.); 5 Servicio de Neumología, Hospital 12 de Octubre, 28041 Madrid, Spain; ady.castro@ciberisciii.es; 6 Servei de Pneumologia (Institut Clínic de Respiratori), Hospital Clínic—Fundació Clínic per la Recerca Biomèdica, Universitat de Barcelona, 08036 Barcelona, Spain; 7 Unidad Médico-Quirúrgica de Enfermedades Respiratorias, Institute of Biomedicine of Seville (IBiS)/Hospital Universitario Virgen del Rocío/CSIC/Universidad de Sevilla, 41012 Sevilla, Spain; 8 Fundació Institut d’Investigació i Innovació Parc Taulí (I3PT), 08208 Sabadell, Spain; 9 Servei de Pneumologia, Hospital Son Espases, Institut d’Investigació Sanitària Illes Balears (IdISBa), Universitat de les Illes Balears, 07120 Palma, Spain

**Keywords:** COPD, sex differences, biomarker discovery, pathophysiology, gut-liver axis

## Abstract

Metabolomic studies in COPD reveal systemic metabolic perturbations, yet sex is often treated as a covariate rather than a biological driver. We aimed to identify plasma metabolites differentiating COPD from controls and to define sex-specific metabolic signatures in both groups. **Methods**: In this controlled observational study (BIOMEPOC cohort), untargeted plasma metabolomics was performed by LC-MS/MS. Differential abundance was tested across four contrasts (COPD vs. controls; men vs. women within controls; men vs. women within COPD; sex-by-disease interaction) with a false discovery rate (FDR) correction. Because smoking history differed between COPD and controls, a post hoc ever-smokers analysis was conducted. **Results**: COPD differed from controls in nine metabolites (all decreased): DL-stachydrine, 3-methyl-L-histidine, fructose, pipecolinic and nipecotic acids, 5-nitro-o-toluidine, conjugated linoleic acid, aminoadipate, and creatinine. This pattern is compatible with metabolic depletion, remodeling, and/or altered flux across multiple compartments rather than simple substrate deficiency, spanning muscle-related pools, amino acid handling, carbohydrate-associated metabolism, and exposome-linked inputs. In ever-smokers, results were directionally consistent, with five metabolites remaining nominally significant. Among controls, five metabolites were higher in men after FDR correction (PABA, cis-4-hydroxy-D-proline, N-acetylasparagine, deoxycarnitine, and creatinine), consistent with physiological sex dimorphism in energy pathways, connective-tissue remodeling, and diet/microbiome-related metabolism. Within COPD, six metabolites differed by sex after FDR correction, defining three axes: creatine energy buffering (men: higher GAA/creatinine, lower creatine), purine/urate handling (men: higher urate), and conjugated bile acids (men: higher GCDCA), implicating muscle bioenergetics, redox/inflammatory tone, and gut–liver crosstalk. **Conclusions**: Plasma metabolomics identifies a pattern compatible with systemic remodeling in COPD and sex-associated divergences in creatine, purine/urate, and bile-acid pathways, supporting a sex-influenced view of systemic COPD heterogeneity and highlighting targets for mechanistic validation.

## 1. Introduction

Chronic obstructive pulmonary disease (COPD) remains a highly prevalent, progressive condition and a leading contributor to chronic morbidity and mortality, with a substantial impact on healthcare utilization and social costs [1,2]. Beyond airflow limitation, COPD is characterized by recurrent exacerbations and frequent systemic comorbidities, which together drive hospitalizations, long-term treatment needs, and indirect costs related to loss of functional capacity and years of life [1,2]. Despite improvements in prevention and management, the clinical and societal burden of COPD remains high and the clinical management is still very generic, reinforcing the need for improving phenotyping and more guided strategies [3,4].

Until very recently, COPD has often been approached as a largely uniform entity. The sex of the patients, in particular, has been treated mainly as a demographic variable rather than a potential determinant in disease biology [5]. However, accumulating clinical evidence suggests that women and men with COPD may differ in symptom burden, structural involvement of the lungs, exacerbation patterns, comorbidity profiles, and outcomes [6,7,8,9,10,11,12]. These observations raise the possibility that COPD involves, at least in part, sex-dependent pathophysiological pathways, potentially shaped by differences in exposures and hormonal milieu. Moreover, metabolomic studies that analyze COPD patients without accounting for sex differences risk masking or diluting pathophysiologically relevant pathways involved in the onset, progression, and clinical expression of the disease.

Metabolomics offers a particularly suitable framework to address this question because the circulating metabolome integrates signals from multiple organs and processes implicated in COPD, including oxidative and nitrosative stress, inflammation, altered bioenergetics, lipid remodeling and host–microbiome interactions [13]. Prior metabolomic studies support the plausibility of sex-specific pathophysiology in COPD, reporting differential involvement of carnitine-related metabolism, nitric oxide/arginine pathways (potentially linked to nitrosative stress), and sphingolipids and ceramide routes [14,15,16]. Moreover, network-based approaches have further suggested sex-dependent metabolite modules that include amino acids, lysophospholipids, bile acids, acylcholines, tricarboxylic acid (TCA) intermediates, xenobiotics and steroid-related signatures [15]. In parallel, growing evidence on the gut–lung axis indicates that microbiome-associated metabolites and bile-acid patterns may contribute to COPD heterogeneity [17,18,19,20] and could plausibly differ by sex.

In the present study, we performed sex-stratified metabolic analyses in COPD patients and controls of similar general characteristics to identify metabolic biomarkers shared across sexes and those that are sex-specific to also evaluate sex-by-disease interaction patterns. We then interpret the resulting signatures in relation to key biological axes, to derive pathophysiological hypotheses that may help explain sex-related differences in COPD.

## 2. Methods

### 2.1. Study Design, Ethics and Participants

This is a controlled observational study embedded within the BIOMEPOC project, a prospective multicenter cohort whose details have been published elsewhere [21]. The study was conducted in accordance with the Declaration of Helsinki and approved by the corresponding institutional ethics committee (ref. 2014/5695/I). Written informed consent was obtained from all participants prior to the study procedure. Participants in the cohort were recruited from participating centers and classified as COPD patients and controls. COPD diagnosis was established on the basis of smoking exposure and post-bronchodilator spirometry demonstrating persistent airflow obstruction (FEV_1_/FVC below the threshold defined in international guidelines). Individuals following specific dietary interventions or receiving formal nutritional support/therapeutic diets were not included. Patients with lung cancer or other chronic respiratory or inflammatory conditions were excluded. Renal dysfunction was defined as the detection at recruitment of mildly abnormal blood urea and creatinine levels, in the absence of a formal diagnosis of kidney disease. All COPD evaluations and biospecimen collection were performed during clinical stability (no exacerbations in the preceding 3 months). Controls were defined as individuals free of any clinically relevant disease and were recruited from the general population. Demographic and clinical variables were obtained using harmonized procedures across centers. Patients and controls for this specific study were randomly chosen from the BIOMEPOC cohort and subsequently selected to ensure a similar distribution of age, sex and nutritional status.

### 2.2. Blood Sampling, Processing and Storage

Peripheral venous blood was collected according to standardized operating procedures to minimize pre-analytical variability. Samples were processed within a predefined time window after collection. Routine laboratory tests (including complete blood count, serum glucose, electrolytes, urea, creatinine, and liver enzymes) were performed prior to the inclusion in the study to avoid major disorders. The main purpose was screening rather than use as study variables, and they were only partially captured in the centralized dataset. In parallel, plasma was obtained from EDTA tubes by centrifugation (1500× *g* for 15 min), aliquoted to avoid repeated freeze–thaw cycles, and stored at −80 °C until metabolomic analysis.

### 2.3. Metabolomics Platform and Quality Control

Untargeted metabolomic profiling was performed using a high-performance liquid chromatography-tandem mass spectrometry (LC-MS/MS) platform at the collaborating metabolomics core facility in Israel (Core Research Facility of Faculty of Medicine, Hebrew University of Jerusalem), following procedures described elsewhere [22,23]. Analytical data acquisition incorporated pooled quality-control samples and blanks at regular intervals to track instrument stability, detect background signals, and monitor batch effects. Metabolites were annotated using high accurate mass and retention-time alignment using Compound Discoverer (v3.3.3.200, Thermo Fisher Scientific, Waltham, MA, USA), with confirmation against an in-house reference library of chemical standards (approximately 450 compounds), following established pipelines. Each identified metabolite intensity was normalized to the total intensity of the sample.

Across all samples, the LC–MS/MS workflow initially detected 2416 features. After quality-control filtering (missingness and technical criteria) and a predefined identification filter, 139 features with at least a Metabolomics Standards Initiative (MSI) annotation level 2 were retained for statistical analyses. Peaks lacking structural annotation or molecular formula (‘unknown’ peaks) were excluded a priori and therefore were not included in inferential testing (including sex-by-disease interaction models) nor in FDR correction.

### 2.4. Statistics of General and Clinical Data

General and clinical data are reported as mean ± standard deviation (SD) or median (interquartile range, IQR) for continuous variables, and counts and percentages for categorical ones. Distributional assumptions for continuous variables were assessed using the Kolmogorov–Smirnov test. Between-group comparisons (patients vs. controls, and men vs. women within each group) were performed using the independent-samples *t* test or the Mann–Whitney U test, when appropriate. All analyses were conducted using SPSS (version 25.0; IBM Corp., Armonk, NY, USA).

### 2.5. Metabolomic Data Preprocessing and Normalization

Raw feature intensities were inspected for data completeness and distributional properties. Features with excessive missingness were filtered using pre-specified thresholds. Remaining intensities were log_2_-transformed to stabilize variance and approximate normality. Normalization was applied to reduce inter-sample technical variability (e.g., scaling to overall signal per sample or an equivalent global approach), and potential outliers were evaluated using distance-based diagnostics and/or principal component patterns.

### 2.6. Differential Abundance and Interaction Analyses

For the metabolomic analysis, we focused on sex-stratified metabolic profiling in COPD and control subjects. The primary comparisons were (I) COPD vs. controls (overall); (II) men vs. women within controls; (III) men vs. women within patients; and (IV) the sex-by-disease interaction, aimed at distinguishing baseline sex differences from COPD-related sex divergence.

Differential metabolite abundance was assessed using regression-based models suited to continuous outcomes. Primary models included terms for disease status, sex, and their interaction (sex × disease) to identify metabolites whose sex effect differed between controls and patients. Covariate adjustment included core demographic and clinical variables (e.g., age and BMI), and additional study design factors (e.g., recruiting center) were incorporated as appropriate for the specific comparison. Multiple testing was controlled using false discovery rate (FDR) correction, with FDR < 0.05 considered statistically significant. Effect sizes were expressed as log_2_ fold changes and/or percentage differences to facilitate clinical interpretability.

Because smoking history differed between controls (consecutively recruited from our general population) and patients, we performed a post hoc analysis restricted to ever-smokers.

### 2.7. Functional Annotation, Pathway Grouping, and Network Representation

Metabolites reaching statistical significance (and selected borderline signals of biological interest) were mapped to biochemical context using curated resources (e.g., HMDB, KEGG, Reactome, PubChem) and classified into functional groups reflecting their predominant metabolic role. In view of pleiotropy, each metabolite was assigned to the most biologically informative category for the purposes of this study, while always acknowledging multi-pathway involvement.

To support interpretation of sex-specific patterns, metabolites were summarized within major biochemical axes relevant to COPD biology (e.g., metabolic pathways related to energy, oxidative stress, purines, bile acids or muscle mass, as well as microbiome-associated co-metabolites). Network visualizations were generated to display metabolite interconnections and to facilitate a comparison between COPD and control networks modulated by sex, while preserving node positions across panels.

## 3. Results

Participant demographic and clinical characteristics are summarized in Table 1. Overall, they were in their seventh decade of life and had overweight-range BMI. As expected, COPD patients and controls differed in smoking exposure and lung function parameters. Within the COPD group, both men and women were predominantly ex-smokers, most patients were classified as GOLD stages 2–3, and their annual exacerbation rate was very low. Comorbidities were prevalent, with cardiovascular abnormalities being the most frequent, followed at a distance by metabolic disorders. The most frequent cardiovascular comorbidity in both controls and COPD patients was arterial hypertension (30% and 49%, respectively), with similar proportions in men and women in both groups. Regarding metabolic comorbidity, the most frequent was type 2 diabetes mellitus (12% in controls and 14% in COPD patients), with a slight, non-significant predominance of men in both groups.

Inhaled bronchodilator use was universal (100%), whereas inhaled corticosteroids (ICS) were used mainly in GOLD group E (with a similar distribution in men and women), and oral corticosteroids (OCS) were used only occasionally in the COPD groups. Only around a half of patients also received diuretic therapy [predominantly thiazide(-type)], mostly because of arterial hypertension (similar proportion for males and females).

### 3.1. COPD vs. Controls (Sex Not Considered)

**Figure 1:**
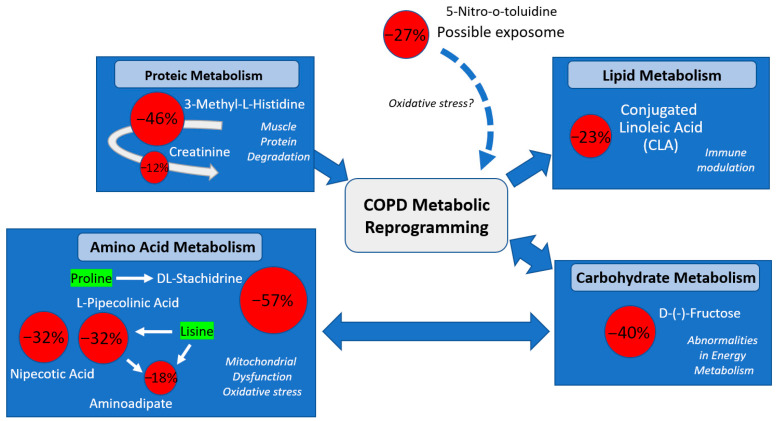
Conceptual summary of metabolic differences between COPD patients and controls. The Figure summarizes the metabolites that were significantly lower in COPD than in controls (FDR-corrected analysis) and their putative biological interpretation. Differential metabolites are grouped into major functional domains (protein/amino acid, carbohydrate and lipid metabolism) and linked to plausible pathophysiological processes, including altered energy metabolism, muscle protein degradation, mitochondrial dysfunction/oxidative stress, immune modulation, and exposome-related influences, consistent with a pattern of systemic metabolic remodeling in COPD.

In the overall comparison between COPD patients and controls, nine metabolites differed significantly after FDR correction (Table 2 and Figure 1). All of them showed lower levels in COPD: DL-stachydrine (−57%), 3-methyl-L-histidine (−46%), D-(-)-fructose (−40%), L(-)-pipecolinic and nipecotic acids (−32% both), 5-nitro-o-toluidine (−27%), 9(Z),11(E)-conjugated linoleic acid (CLA) (−23%), aminoadipate (−18%), and creatinine (−12%).

Given the relative imbalance in smoking history, we repeated the analysis in ever-smokers only. This analysis showed broadly similar results, although the reduced number of subjects in each group meant that only five of the nine former differential metabolites reached nominal significance [3-methyl-L-histidine, L(-)-pipecolinic and nipecotic acids, 5-nitro-o-toluidine, and aminoadipate). By contrast, four additional metabolites reached nominal statistical significance: sarcosine, metformin (consistent with treated diabetes in this subset), ethyl-D-glucuronide and L-methionine sulfoxide.

### 3.2. Sex Differences Among Controls

**Figure 2:**
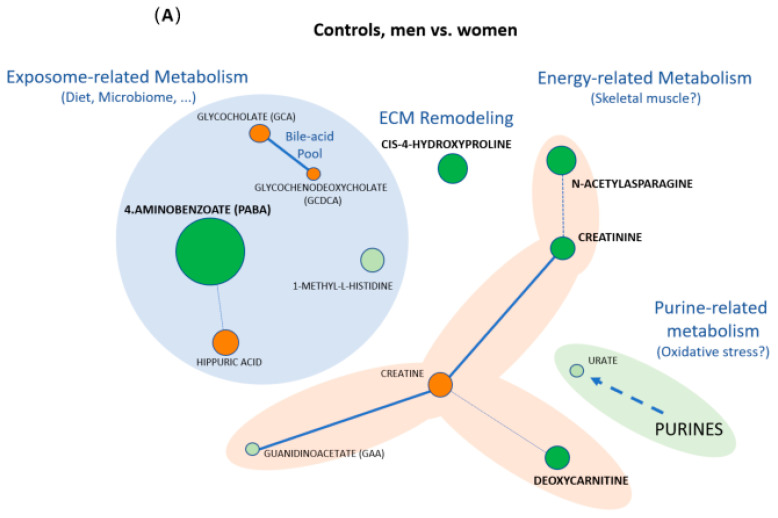

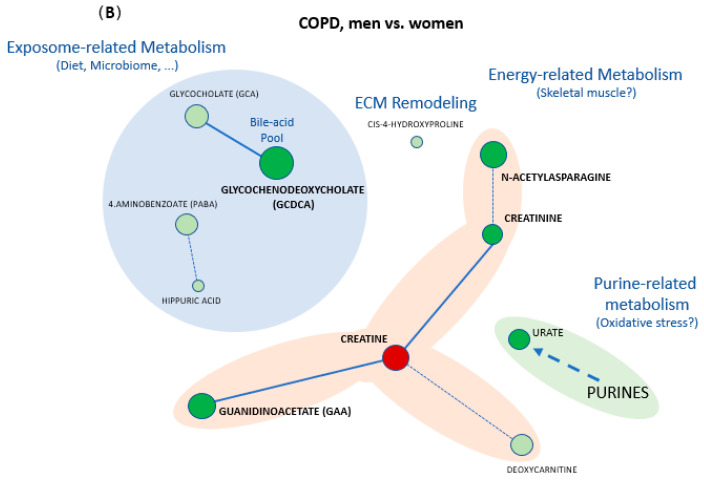
Sex-related plasma metabolomic differences in controls and COPD. Panel (**A**) shows the comparison men vs. women in controls, and panel (**B**) the same comparison in COPD. Each node represents one metabolite. Node color indicates the direction and statistical significance of the sex differences: dark green, higher in men with FDR < 0.05; light green, higher in men but not statistically significant (FDR ≥ 0.05); dark red, higher in women with FDR < 0.05; light red, higher in women but not statistically significant (FDR ≥ 0.05). Node size is proportional to the absolute percent difference between sexes. Lines connect metabolites according to biological relatedness: solid lines denote well-established biochemical relationships within a pathway, whereas dashed lines indicate functional or contextual associations and are included to facilitate biological interpretation.

Within Controls, five metabolites showed significant differences between men and women after FDR correction, all with higher levels in men: 4-aminobenzoate (PABA) (374%), cis-4-hydroxy-D-proline (53%), N-acetylasparagine (51%), deoxycarnitine (26%), and creatinine (25%) (Table 2, Figure 2A). Three additional metabolites reached nominal significance but did not remain significant after FDR adjustment: 1-methyl-L-histidine (26%), creatine (−29%) and hippuric acid (−41%).

### 3.3. Sex Differences Among COPD Patients

Among patients, six metabolites differed significantly between men and women after FDR correction (Table 2, Figure 2B). Five were higher in men: glycochenodeoxycholate (GCDCA) (86%), guanidinoacetate (GAA) (47%), N-acetylasparagine (45%), creatinine (26%) and urate (21%). In contrast, creatine was lower (−43%). Deoxycarnitine in turn showed a nominally significant sex difference (25%) but did not remain significant after FDR correction.

### 3.4. Sex-by-Disease Integrated Analysis

We tested sex-by-group (COPD vs. control) interaction terms for each metabolite to assess whether disease-associated differences varied by sex. Nominal interactions are reported as exploratory findings in Table 2; however, after FDR correction, no interaction terms reached statistical significance.

## 4. Discussion

Four main observations emerged from this sex-stratified metabolomic study. First, when sex was not considered, COPD patients showed lower circulating levels of nine metabolites compared with controls. Second, among controls, men exhibited higher levels of five metabolites, consistent with expected baseline sex-related differences. Third, within COPD, six metabolites differed between men and women, with a pattern that included higher GCDCA, GAA, N-acetylasparagine, creatinine and urate in men, together with lower creatine. Finally, an integrated sex-by-disease exploration suggested that some sex differences may be amplified, attenuated or even inverted in COPD relative to controls. However, these interaction signals did not remain significant after FDR correction and therefore, should be regarded as hypothesis-generating.

### 4.1. COPD Versus Controls

Previous metabolomic studies consistently depict COPD as a systemic “catabolic-inflammatory” condition, with a reproducible fingerprint of lipid remodeling (especially fatty acids, glycerophospholipids/sphingolipids, eicosanoid-related mediators and acylcarnitines), together with shifts in amino acid pathways and energy intermediates consistent with muscle catabolism, mitochondrial stress and altered oxidative efficiency [13,15,24,25,26,27,28,29]. In a very recent plasma metabolomics study from our group, these patterns were again dominated by lipids (showing higher short/medium-chain fatty acids and multiple acylcarnitines, with lower long/very-long-chain fatty acids). This was complemented by perturbations in amino acids and carbohydrate-related metabolites, yielding a multi-metabolite signature with strong discriminatory performance [30]. Proteomic work from our group reinforces this systemic component by identifying a core COPD-associated inflammatory signature and broader dysregulation of innate/adaptive immunity pathways [31].

The present study, characterized by the untargeted identification of metabolites (i.e., wider spectrum than in our previous targeted study), adds a coherent set of signals that align COPD with combined metabolic stress and systemic inflammation (Figure 1). Prior proteomic/metabolomic reports have pointed to disturbed creatinine-related pathways and reductions in amino acid-linked metabolites such as aminoadipate [27,32,33], while other cohorts have shown increased 3-methyl-L-histidine in specific clinical contexts, likely reflecting myofibrillar protein breakdown [28,34]. In parallel, carbohydrate handling appears relevant, as fructose and fructose-related metabolites have been linked to COPD risk, prognosis, and specific exposures such as exacerbations or exercise [35,36,37,38]. Finally, our findings in polyunsaturated fatty acids (PUFA)-related biology (including linoleic-associated changes) fit with the literature [39,40,41,42], connecting dietary and microbiome-related PUFA patterns with systemic inflammation/oxidative stress and clinical outcomes in COPD [43,44], whereas the detection of less-characterized diet/microbiome-associated metabolites (e.g., DL-stachydrine) supports the idea that exogenous inputs and host–microbiome interactions may contribute to the circulating COPD metabolic phenotype [45].

In this context, the fasting COPD metabolite signature observed here is compatible with systemic metabolic depletion and/or remodeling. Notably, all nine metabolites differentiating COPD from controls were decreased in patients. Because samples were obtained under fasting conditions, the differences are unlikely to reflect acute dietary intake; however, contributions from habitual diet cannot be fully excluded. Moreover, the observed differences between patients and controls cannot be specifically attributed to reduced lean mass, as opposed to altered turnover or renal handling, without body-composition data in the control group.

Lower creatinine and 3-methyl-L-histidine may reflect reduced lean mass-related metabolic pools and altered protein turnover [46,47], consistent with the recognized systemic involvement in COPD, and more specifically with muscle dysfunction and sarcopenia (compatible with *sarcopenic obesity* in this case) [48]. However, creatinine is a surrogate marker of muscle-associated metabolism rather than a direct measure of lean mass. Importantly, because direct measures of body composition (e.g., FFMI) were not available in our controls, we cannot determine whether these findings primarily capture reduced muscle mass, altered turnover, or other contributors such as renal handling. Nonetheless, renal function was preserved in most of the individuals included in our study and the directionality of changes is consistent with a diminished muscle-associated metabolic signal.

The coordinated reduction in amino acid-related metabolites, including compounds linked to lysine metabolism (aminoadipate, pipecolinic and nipecotic acids), suggests a shift in amino acid handling that may accompany chronic disease states and sustained inflammatory–oxidative burden [33,49]. In parallel, lower fructose points to an altered carbohydrate-associated flux [37], although the mechanistic interpretation of fructose alone should be necessarily cautious without parallel glucose/insulin phenotyping [50]. Finally, decreases in DL-stachydrine and conjugated linoleic acid (CLA), together with a reduction in 5-nitro-o-toluidine (a xenobiotic compound), indicate that COPD is associated with differences in metabolites often influenced by diet, the gut microbiome, and/or environmental exposures [51,52,53]. This is relevant because COPD is closely tied to cumulative exposures, and many circulating metabolites integrate the host metabolism with exposome-related inputs.

Finally, a recent and very wide study has described a Metabolic Vulnerability Index (MVI) based on six plasma metabolomic biomarkers expressing inflammation and metabolic malnutrition [glycoprotein acetyls (GlycA), small high-density lipoprotein particles (sHDL), citrate, and branched-chain amino acids (isoleucine, leucine, and valine) that are directly associated with COPD risk and inversely with lung function, independently of traditional risk factors [54].

A key issue in interpreting the COPD-control contrasts is the marked difference in lifetime smoking exposure between groups. COPD patients were predominantly ex-smokers and had a much higher cumulative tobacco exposure, whereas controls also included never-smokers and had low pack-years overall. Accordingly, some of the observed COPD-associated differences could reflect persistent metabolic consequences of heavy smoking rather than COPD per se, a limitation shared by many COPD cohorts. In this regard, several authors have reported that metabolomic signatures differ between smokers with and without COPD [14,55,56], particularly among women [16]. Reassuringly, our post hoc analysis restricted to ever smokers, together with models adjusted for pack-years, are broadly consistent with the main findings, though underpowered to definitively exclude smoking effects. Moreover, the nominal significance of the four newly differential metabolites is consistent with a potential contribution of oxidative stress and exposome-related mechanisms in disease pathophysiology.

However, we acknowledge the possibility of residual confounding when interpreting some findings as attributable solely to COPD, since cumulative tobacco exposure was, as expected, substantially higher in patients than in a control group representative of the general population without any clinically relevant disease. In this context, certain metabolites, such as stachydrine and 5-nitro-o-toluidione, may partly reflect the persistent consequences of a persistent smoking legacy rather than COPD-specific biology.

### 4.2. Sex-Related Differences in Controls

Our results suggest that the main differences between control men and women were related to energy metabolism, connective tissue, and interaction with the exposome (diet and microbiome) (Figure 2A). In this population, five metabolites were higher in men than in women after FDR correction. Their pattern is consistent with the expected baseline sexual dimorphism in systemic metabolism.

Creatinine is a well-known proxy of lean mass and typically differs by sex even when BMI is similar [57,58]. The concomitant increase in deoxycarnitine may reflect sex-related differences in carnitine availability (a vitamin-like quaternary amine essential for mitochondrial long-chain fatty acid transport) and in fatty-acid-linked bioenergetics, potentially related to body composition and metabolic regulation [59,60]. Although the women in this cohort were mostly in their seventh decade, and thus largely postmenopausal, sex-related differences in lipid and energy metabolism may persist due to long-term endocrine history and potential (not confirmed in our study) differences in body composition.

Higher cis-4-hydroxy-D-proline in men is compatible with differences in collagen-related turnover and connective-tissue remodeling, which again may reflect baseline differences in musculoskeletal composition and/or physical activity, which have been reported to be predominantly lower in females [58,61,62]. The higher levels showed by men in N-acetylasparagine (an acetylated amino acid) relative to women may reflect a combination of differences in acetyl-CoA-dependent metabolism, amino acid handling, and/or renal clearance [63]. The latter can be reasonably minimized because renal function was preserved in all our controls. Although more subtle variations in kidney function cannot totally be ruled out, we believe that this sex-related difference is an actual surrogate of divergences in metabolism. The marked elevation of PABA in men, in turn, suggests a strong contribution from diet- and/or microbiome-related variability, given the known links of aromatic metabolites to nutrient intake and microbial folate-related metabolism [64,65].

Finally, three additional metabolites showed nominal sex differences in controls. The higher 1-methyl-L-histidine and lower hippurate levels observed in men are also biologically coherent with the axes described above: the former metabolite is often linked to dietary protein patterns and is less likely to reflect muscle-related metabolism (where 3-methylhistidine is the classical marker), whereas the latter is a classic gut microbial–host co-metabolite influenced by diet and hepatic conjugation [66,67,68]. Although not definitive, these trends support the plausibility that baseline sex differences in our controls extend to these metabolic domains.

These findings are consistent with the broader literature showing substantial sex-related differences in systemic metabolism. However, the menstrual cycle can alter the level of specific metabolites in women of reproductive age [69], and even the same diet may elicit different metabolic responses in men and women [70,71]. In any case, major differences have been reported in plasma lipids between men and women (reaching 70–80% of measured lipids species in some studies) [72,73]. In general, women show higher levels of fatty acids than men. Interestingly, this is the particular case of long-chain polyunsaturated fatty acids (PUFA) such as arachidonic and docosahexaenoic acids (ARA and DHA, respectively), but not necessarily their precursors linoleic and α-linoleic acids (LA and ALA, respectively) [74]. One exception may be acylcarnitine, belonging to fatty esters, some of which (as well as occurs with fatty acids) are lower in women [75]. The latter also appear to have higher levels of phosphatidylcholines (PCs) and sphingomyelins (SMs) than men [72,73,76], particularly in adulthood. Reported differences in ceramides (Cer), often higher in women [72,77], may depend on sex-specific fatty-acid composition. Conversely, men have been reported to show higher lysophospholipids and triacylglycerides (TAG) [72,73,76,78], although these differences may attenuate in older individuals. Some authors have suggested that these sex-related lipid differences are partially driven by estrogen effects, while other factors such as diet and lifestyle may also contribute [78].

Protein and amino acid metabolism also show notable sex differences. Branched-chain amino acids (BCAA) (e.g., leucine, isoleucine and valine) are often higher in men, whereas other amino acids (e.g., glycine) show the opposite pattern [79,80,81]. Higher BCAA levels in men have been related to their greater muscle mass, but they may also indicate relatively higher insulin resistance and/or increased protein catabolism. In turn, higher glycine in women could relate to differences in oxidative-stress handling, given its role as a substrate for glutathione (GSH). Regarding carbohydrates, men often show higher levels of glucose, mannose, and arabitol [80], while the first two may again relate to sex differences in body composition and insulin sensitivity, arabitol may reflect both diet and microbiome-related inputs. Metabolites more directly linked to energy metabolism also differ between sexes: phosphate (Pi) and acetylphosphate have been reported to be higher in women, whereas succinylcarnitine and malate are higher in men [80,81]. This pattern supports the concept that men and women handle energy flux and mitochondrial metabolism differently. In addition, sex differences in Pi may also reflect differences in phospho-calcium metabolism, particularly in postmenopausal age. Conversely, higher succinylcarnitine and malate in men is compatible with greater substrate flux toward the TCA cycle, potentially combined with differences in oxidative efficiency.

Another relevant domain is the gut microbiome, which also shows sex-related differences. While early work suggested a broadly similar gut microbiota composition between sexes [82], more recent studies report that women generally exhibit higher Akkermansia and Bifidobacterium, whereas men show increased Prevotella and Escherichia, among other differences that may influence circulating metabolites even in healthy individuals [83,84]. Moreover, the microbiota may interact with sex hormones, potentially generating additional sex-dependent changes in plasma metabolites [83,85], supporting the concept of “microgenderome” [82].

### 4.3. Sex-Specific Metabolic Signatures in COPD and Pathophysiological Implications

Within COPD, sex differences involved six metabolites that naturally cluster into three biologically interpretable axes (Figure 2B): (I) the creatine-GAA-creatinine system and related muscle energy metabolism, (II) purine-related metabolism (with urate as the final product of purine degradation), and (III) conjugated bile acids and the gut–liver axis.

Axis I. COPD men exhibited higher GAA and creatinine but lower circulating creatine compared with women, alongside higher N-acetylasparagine. Considered together, these findings point to sex-related differences in the creatine system, which is central to rapid energy buffering and transfer through the ATP-phosphocreatine/creatine kinase pathway and is closely linked to skeletal muscle metabolic pools [86,87]. This combination could reflect divergences in creatine biosynthesis and handling (i.e., AGAT/GAA- and GAMT-dependent synthesis, utilization, and compartmentalization), potentially shaped by muscle metabolic adaptation in COPD and/or underlying differences in body composition [88,89,90]. Finally, the nominal tendency toward higher deoxycarnitine in men is directionally consistent with a broader energy-metabolism component and may indicate sex differences in carnitine-related bioenergetic pathways in COPD, given that deoxycarnitine (γ-butyrobetaine) is the immediate precursor of L-carnitine and that carnitine/acylcarnitine signatures show sex-specific associations in COPD cohorts [15,91].

Axis II. Urate was higher in men with COPD. Uric acid (the end product of purine catabolism) may rise with increased purine turnover and has been linked to oxidative stress, systemic inflammation, and hypoxemia, all of which are relevant to COPD pathobiology [92,93]. However, circulating urate is also strongly shaped by renal handling and by common clinical modifiers, including diuretic therapy and cardiometabolic comorbidities [94,95]. Although renal function was preserved in our patients, even within the reference range subtle inter-individual variability in renal handling may still influence metabolites such as urate. Cardiovascular comorbidities were relatively frequent, and approximately half of participants of both sexes were receiving diuretics (mainly for arterial hypertension). Because diuretic therapy is a well-known modifier of circulating urate, this is an important consideration when interpreting the purine–urate axis. However, diuretic therapy was broadly similar in men and women, whereas men consistently showed higher plasma urate levels, with a more pronounced difference in COPD patients. This pattern supports the interpretation of a sex-associated divergence in the purine–urate axis in COPD rather than an effect driven solely by diuretic exposure. Nevertheless, residual confounding related to the overall comorbidity burden or unmeasured aspects of diuretic exposure (e.g., class or dose) cannot be fully excluded.

Axis III. Although we did not observe a global bile-acid panel shift, the isolated GCDCA signal in men with COPD, together with the absence of biochemical evidence of cholestasis, may point to alterations in enterohepatic circulation, bile-acid transport and signaling, and/or microbiome-mediated bile-acid transformation [96,97,98]. Smoking can shape gut microbiome composition and secondary microbial bile-acid conversions, which may plausibly influence circulating conjugated bile acids such as GCDCA. Bile acids are increasingly recognized as metabolic and immunomodulatory mediators that integrate hepatic metabolism with gut microbial ecology and systemic inflammatory tone [96,99,100]. They act through the farnesoid X receptor and the Takeda G protein-coupled receptor 5 (FXR and TGR5, respectively), among others, thereby regulating bile-acid homeostasis while also influencing lipid and glucose metabolism and modulating inflammatory pathways and oxidative stress [96,101,102]. Collectively, these observations are consistent with the hypothesis that gut–liver metabolic crosstalk, potentially shaped by host–microbiome interactions in bile-acid metabolism, may contribute to sex-specific systemic features in COPD. However, because we did not measure gut microbiota or bile-acid transport/signaling directly, this interpretation remains hypothesis generating.

Collectively, these axes align with prior metabolomics studies suggesting that women and men with COPD may show differential engagement of amino acid- and nitric oxide-related metabolism versus lipid-related pathways, and that metabolite-network modules involving amino acids, bile acids, and xenobiotics can be sex dependent [13]. Our findings add to this literature by identifying a small set of robust sex-associated metabolites in COPD and by pointing to creatine-related energy buffering, purine/urate handling, and bile-acid biology as candidate domains underlying sex-specific systemic patterns.

Across metabolic studies, sex clearly modulated the systemic (and airway) metabolic signature of COPD, suggesting partially distinct biological “routes” to a shared clinical syndrome, which may contribute to differences in clinical expression. It is well established that women with COPD are often more symptomatic, report worse health-related quality of life for a similar degree of airflow limitation, may experience a steeper lung-function decline, show higher airway hyperresponsiveness, and exhibit sex-related differences in structural changes affecting airways, lung parenchyma and skeletal muscles, exacerbation burden, and even mortality [6,10,11,103,104,105]. A robust and replicated observation is that circulating carnitines are lower in women with COPD, both versus healthy women and versus men with COPD, while several reports also indicate that oxidative stress-related markers are more pronounced in COPD females [16], with dysregulation in these pathways (including the autotaxin–lysophosphatidic acid axis) and probable downregulation of antioxidant genes [16,106]. In keeping with this, women with COPD also show stronger signals in pathways connected to nitric oxide/arginine metabolism, a major source of reactive nitrogen species (RNS) [16], together with higher estimates of lipid β-oxidation, purine degradation and endocannabinoid production, lower carnitine levels but a higher carnitine/acetylcarnitine ratio in some datasets [15,16,107]. By contrast, male COPD tends to display a greater involvement of lipid remodeling (fatty acids, sphingolipids and ceramides) and abnormalities in tryptophan metabolism relative to women with the disease [15,108], potentially relating to sex-dependent host–microbiome interactions. Moreover, sex-specific network analyses place greater weight in men on modules combining amino acids with lysophospholipids and derivatives (e.g., lysophosphatidic acid, LPA), bile acids and acylcholines, or amino acids with TCA intermediates and xenobiotics, whereas steroid-centered mediators are more specific to women [14,15,16,107]. Overall, these patterns are commonly interpreted as the combined result of hormonal influences and a sex-related, differentiated exposome shaped by cultural and environmental factors [6].

### 4.4. Sex-by-Disease Interaction: Baseline vs. COPD-Related Divergence

The integrated sex-by-disease analysis aimed to explore whether baseline sex differences observed in controls were modified in COPD. In this exploratory framework, several metabolites (including conjugated bile acids and hippurate) showed nominal patterns suggestive of sex-by-group effects, and some metabolites displayed strong sex differences in controls but weaker differences in COPD, potentially consistent with attenuation of physiological dimorphism in patients. However, none of the interaction terms remained significant after FDR correction. Accordingly, these observations should be considered hypothesis-generating and not over-interpreted; they mainly motivate future studies in larger, well-characterized cohorts to formally test sex-by-disease interactions with adequate statistical power.

### 4.5. Strengths and Limitations

This study has several strengths, including fasting sampling, use of a consistent metabolomics platform, and a sex-stratified analytical strategy that distinguishes baseline (physiological) sex differences from those observed within COPD.

Nevertheless, limitations should be acknowledged. First, statistical power may have been limited by subgroup sample sizes, particularly for sex-by-disease interaction testing, which is inherently underpowered relative to main effects. Therefore, interaction results should be considered exploratory. Second, detailed body-composition measures were not available in controls, limiting mechanistic interpretation of patient-control differences in muscle-related metabolites. This limitation mainly affects the COPD-control contrast, whereas within-COPD sex comparisons are less impacted because FFMI was available in most patients. In addition, physical activity was not assessed; thus, residual confounding by activity-related factors cannot be excluded. Routine biochemical laboratory data were also not uniformly recorded in the centralized dataset across centers, as they were used primarily for eligibility screening rather than as study variables; therefore, residual confounding by incompletely captured biochemical abnormalities remains possible.

Differences in lifetime tobacco exposure between COPD patients and controls may also confound the COPD-control contrast; however, a post hoc analysis restricted to ever-smokers in both groups showed directionally similar results. Some metabolites may be influenced by habitual diet, microbiome variability, medication use, and comorbidities. While key covariates were accounted for, residual confounding by incompletely measured factors remains possible.

Finally, hormone concentrations were not measured, and the use of hormone replacement therapy (HRT) or androgen therapy was not systematically captured in the available clinical dataset. Although such therapies were uncommon in this age range and clinical context in the participating centers, unrecorded use in individual participants cannot be excluded. Therefore, more detailed hormonal profiling would have strengthened the interpretation of sex-associated metabolomic differences. We emphasize that the analyses and interpretations presented in this manuscript refer to sex-associated metabolic patterns and are not intended to infer sociocultural gender effects.

## 5. Conclusions

In summary, metabolomics identified a set of metabolites decreased in COPD relative to controls and revealed distinct sex-associated signatures within both controls and COPD. In patients, sex differences involved the creatine system, purine/urate-related metabolism, and conjugated bile acids, highlighting biological domains that may contribute to sex-dependent systemic manifestations of COPD. While sex-by-disease interaction patterns were exploratory, the overall results support a sex-informed view of COPD heterogeneity and provide mechanistically plausible hypotheses to guide future validation in larger cohorts with detailed exposure and body-composition phenotyping.

## Figures and Tables

**Table 1:** Sociodemographic and clinical characteristics of study participants.

	CONTROL (N = 64)	CONTROL		COPD (N = 88)	COPD	
		Men	Women		Men	Women
		(N = 27)	(N = 37)		(N = 46)	(N = 42)
**Sociodemographic Data**						
Age, mean ± SD, yr.	63 ± 9	62 ± 8	63 ± 11	64 ± 7	64 ± 6	65 ± 8
BMI, mean ± SD, (kg/m^2^)	27.9 ± 4.3	28.7 ± 4.5	28.3 ± 4.1	27.4 ± 6.1	27.8 ± 5.0	27.1 ± 6.9
FFMI, mean ± SD (kg/m^2^)	---	---	---	16.4 ± 2.7	18.2 ± 3.0	14.6 ± 2.1 ^xxx^
**Lung Function**						
Post BD FEV_1_ (% pred.)	97 ± 15	96 ± 17	97 ± 14	48 ± 18^###^	48 ± 18 ***	48 ± 18 ***
FEV_1_/FVC (%), mean ± SD	78 ± 5	78 ± 6	78 ± 5	47 ± 12^###^	46 ± 12 ***	48 ± 12 ***
DLco (% pred.), mean ± SD	92 ± 4	93 ± 11	92 ± 10	47 ± 18^###^	49 ± 19 ***	43 ± 16 ***
**Relevant Events**						
Exacerbations, median (IQR)	---	---	---	1 (0.8–2.3)	1 (0.1–2.3)	1 (0.9–2.8)
Hospitalizations, median (IQR)	---	---	---	0 (0.2–1.3)	0 (0.3–1.9)	0 (0.2–1.1)
**GOLD stage, N (%)**						
1	---	---	---	7 (8)	4 (9)	3 (7)
2	---	---	---	26 (29)	13 (27)	13 (31)
3	---	---	---	43 (48)	23 (49)	20 (48)
4	---	---	---	13 (15)	7 (15)	6 (14)
**GOLD group, N (%)**						
A	---	---	---	13 (15)	6 (13)	7 (17)
B	---	---	---	19 (21)	11 (23)	8 (19)
E	---	---	---	57 (64)	30 (64)	27 (64)
**Smoking status, N (%)**						
Smoker	7 (11)	3 (11)	4 (11)	38 (43) ^###^	19 (41) ***	19 (45) ***
Ex-smoker	24 (38)	16 (59)	8 (22)	50 (57) ^###^	27 (59)	23 (55) ***
Never smoker	33 (51)	8 (30)	25 (67)	-	-	-
Pack-years, mean ± SD	9 ± 14	15 ± 15	5 ± 11	49 ± 21 ^###^	47 ± 16 ***	50 ± 24 ***
**COPD treatments**						
Inhaled bronchodilators, %	---	---	---	100	100	100
Inhaled corticosteroids, %	---	---	---	55	48	62
Oral corticosteroids, %	---	---	---	6	4	7
Xanthines, %	---	---	---	5	2	3
Roflumilast, %				6	3	3
**Comorbidities**						
Charlson index, mean ± SD	---	---	---	2.85 ± 1.51	2.98 ± 1.80	2.72 ± 1.09
Cardiovascular (%)	---	---	---	39.5	42.9	35.9
Metabolic (%)	---	---	---	14.0	15.6	12.2
Renal dysfunction (%)	---	---	---	6.7	7.1	6.3

Values are expressed as median (IQR) or mean ± SD for continuous variables, and N (percentage) for categorical variables. ^###^, *p* < 0.001 COPD vs. controls. ***, *p* < 0.001 COPD men or COPD women vs. their respective controls; ^xxx^, *p* < 0.001 COPD women vs. COPD men. **Abbreviations**: COPD, chronic obstructive pulmonary disease; BMI, body mass index; BD, bronchodilator; FFMI, fat free mass index; FEV_1_, forced expiratory volume in 1 s; FVC, forced vital capacity; DLco, diffusing capacity of the lungs for carbon monoxide; exacerbations, Nº in the previous year; hospitalizations, Nº in the previous year; GOLD, global initiative for obstructive lung disease.

**Table 2:** Differential plasma metabolites by disease status and sex, and sex-by-disease interaction.

	Nominal *p* Value	FDR *p* Value	Log2FC	Δ (%)
**COPD vs. Control**				
3-Methyl-L-histidine	**9.4966 × 10^−5^**	**0.01248836**	−0.89972918	−46
DL-Stachydrine	**0.00017969**	**0.01248836**	−1.22828573	−57
L(-)-Pipecolinic acid	**0.00043610**	**0.01714131**	−0.56396313	−32
Nipecotic acid	**0.00053486**	**0.01714131**	−0.55234167	−32
Creatinine	**0.00073816**	**0.01714131**	−0.18851814	−12
5-Nitro-o-toluidine	**0.00073991**	**0.01714131**	−0.45658021	−27
D-(-)-Fructose	**0.00134300**	**0.02666822**	−0.73747279	−40
Aminoadipate	**0.00172987**	**0.03005656**	−0.29385630	−18
9(Z), 11(E)-Conjugated linoleic acid (CLA)	**0.00321460**	**0.04964764**	−0.37789357	−23
**CONTROL, Men vs. Women**				
N-Acetylasparagine	**2.2461 × 10^−6^**	**0.00031221**	0.59627675	51
Creatinine	**0.00013438**	**0.00933971**	0.31841966	25
CIS-4-Hydroxy-D-proline	**0.00074320**	**0.03442474**	0.60959008	53
4-Aminobenzoate (PABA)	**0.00122703**	**0.03442474**	2.24525338	374
Deoxycarnitine	**0.00123830**	**0.03442474**	0.32986919	26
1-Methyl-L-histidine	**0.00672832**	0.15587289	0.33518994	26
Creatine	**0.01750578**	0.18717715	−0.49989277	−29
Hippuric acid	**0.03030712**	0.30090637	−0.77166479	−41
Glycochenodeoxycholate (GCDCA)	0.66748001	0.95105924	−0.15950536	−10
Guanidinoacetate (GAA)	0.22113197	0.69857599	0.16473682	12
Urate	0.17111886	0.64285195	0.16696698	12
Glycocholate (GCA)	0.14831595	0.61180130	−0.33550142	−21
**COPD, Men vs. Women**				
Creatine	**4.4143 × 10^−6^**	**0.00061359**	−0.80131632	−43
Creatinine	**3.7486 × 10^−5^**	**0.00234747**	0.33089770	26
Guanidinoacetate (GAA)	**5.0665 × 10^−5^**	**0.00234747**	0.55773848	47
N-Acetylasparagine	**0.00031134**	**0.01081905**	0.53373336	45
Urate	**0.00182617**	**0.04526592**	0.27275703	21
Glycochenodeoxycholate (GCDCA)	**0.00195392**	**0.04526592**	0.89276318	86
Deoxycarnitine	**0.03572152**	0.39646855	0.32222859	25
Glycocholate (GCA)	0.05825312	0.52133380	0.49769514	41
CIS-4-Hydroxy-D-proline	0.48244042	0.86736708	0.11864357	9
4-Aminobenzoate (PABA)	0.53912718	0.89212711	0.35625671	28
Hippuric acid	0.79106152	0.96119854	0.08957544	6
1-Methyl-L-histidine	0.97331894	0.98721720	−0.00462846	0
**Δ/Σ Control-COPD** **Men vs. Women**				
Glycochenodeoxycholate (GCDCA)	**0.02286252**	0.98287637	1.03116749	104
Glycocholate (GCA)	**0.02795748**	0.98287637	0.80356493	75
CIS-4-Hydroxy-D-proline	**0.02799958**	0.98287637	−0.52752013	−31
1-Methyl-L-histidine	**0.03154244**	0.98287637	−0.41247400	−25
Hippuric acid	**0.03952411**	0.98287637	1.03116859	104
Guanidinoacetate (GAA)	**0.04324125**	0.98287637	0.38106911	30
4-Aminobenzoate (PABA)	0.14875442	0.98287637	−1.29431586	−59
Creatine	0.20221863	0.98287637	−0.31473527	−20
N-Acetylasparagine	0.42108002	0.98287637	−0.15292503	−10
Urate	0.50864116	0.98287637	0.09077780	6
Deoxycarnitine	0.89104642	0.98287637	0.02647081	2
Creatinine	0.96478498	0.98287637	0.00464836	0

Nominal *p* value, unadjusted two-sided *p* value; FDR, false discovery rate-adjusted *p* value; Log2FC, the log2-transformed fold change for the contrast indicated (positive values denote higher levels in the first group listed, negative values denote higher values in the second group). Δ (%), the corresponding percent difference deriving from Log2FC. Δ/Σ Control-COPD (Men vs. Women), summarizes the sex effect modification by disease status (differences in sex contrasts between controls and COPD); values are reported as interaction estimates in the same Log2FC and Δ (%) units.

## Data Availability

The original contributions presented in this study are included in the article. Further inquiries can be directed to the corresponding author.

## Appendix A

Members of the BIOMEPOC group (in alphabetical order): Mireia Admetlló, Alvar Agustí, Carlos Alvarez-Martínez, Esther Barreiro, Oswaldo Antonio Caguana, Carme Casadevall, Ferran Casals, Robert Castelo, Ady Castro-Acosta, Rocío Córdova, Borja G. Cosío, Rosa Faner, Laura I Furlong, Marian García, José G. González-García, Carmen Hernández-Carcereny, José Luis López-Campos, Eduardo Márquez, Eduard Monsó, Concepción Montón, Miren Josune Ormaza, Alexandre Palou, Sergi Pascual, Germán Peces-Barba, Pau Puigdevall, Diego A. Rodríguez-Chiaradia, Ferran Sanz, Luis Seijo, Montserrat Torà, Yolanda Torralba, and Carles Vilaplana.
